# Designing and implementing sample and data collection for an international genetics study: the Type 1 Diabetes Genetics Consortium (T1DGC)

**DOI:** 10.1177/1740774510373497

**Published:** 2010-08

**Authors:** Joan E Hilner, Letitia H Perdue, Elizabeth G Sides, June J Pierce, Ana M Wägner, Alan Aldrich, Amanda Loth, Lotte Albret, Lynne E Wagenknecht, Concepcion Nierras, Beena Akolkar, the T1DGC

**Affiliations:** ^a^Department of Biostatistics, School of Public Health, University of Alabama at Birmingham, Birmingham, AL, USA, ^b^Division of Public Health Sciences, Wake Forest University Health Sciences, Winston Salem, NC, USA, ^c^Hagedorn Research Institute, Gentofte, Denmark, ^d^Department of Endocrinology, Hospital Universitario Insular de Gran Canaria, Las Palmas de Gran Canaria, Spain, ^e^Department of Medical and Surgical Science, Universidad de Las Palmas de Gran Canaria, Las Palmas de Gran Canaria, Spain, ^f^University of Alaska Anchorage College of Arts and Sciences, Integrated Sciences, Anchorage, AK, USA, ^g^Burnet Clinical Research Unit, Walter & Eliza Hall Institute of Medical Research, Melbourne, Australia, ^h^Juvenile Diabetes Research Foundation International, New York, NY, USA, ^i^Division of Diabetes, Endocrinology and Metabolic Diseases, National Institute of Diabetes and Digestive and Kidney Diseases, National Institutes of Health, Bethesda, MD, USA

## Abstract

***Background and Purpose*** The Type 1 Diabetes Genetics Consortium (T1DGC) is an international project whose primary aims are to: (a) discover genes that modify type 1 diabetes risk; and (b) expand upon the existing genetic resources for type 1 diabetes research. The initial goal was to collect 2500 affected sibling pair (ASP) families worldwide.

***Methods*** T1DGC was organized into four regional networks (Asia-Pacific, Europe, North America, and the United Kingdom) and a Coordinating Center. A Steering Committee, with representatives from each network, the Coordinating Center, and the funding organizations, was responsible for T1DGC operations. The Coordinating Center, with regional network representatives, developed study documents and data systems. Each network established laboratories for: DNA extraction and cell line production; human leukocyte antigen genotyping; and autoantibody measurement. Samples were tracked from the point of collection, processed at network laboratories and stored for deposit at National Institute for Diabetes and Digestive and Kidney Diseases (NIDDK) Central Repositories. Phenotypic data were collected and entered into the study database maintained by the Coordinating Center.

***Results*** T1DGC achieved its original ASP recruitment goal. In response to research design changes, the T1DGC infrastructure also recruited trios, cases, and controls. Results of genetic analyses have identified many novel regions that affect susceptibility to type 1 diabetes. T1DGC created a resource of data and samples that is accessible to the research community.

***Limitations*** Participation in T1DGC was declined by some countries due to study requirements for the processing of samples at network laboratories and/or final deposition of samples in NIDDK Central Repositories. Re-contact of participants was not included in informed consent templates, preventing collection of additional samples for functional studies.

***Conclusions*** T1DGC implemented a distributed, regional network structure to reach ASP recruitment targets. The infrastructure proved robust and flexible enough to accommodate additional recruitment. T1DGC has established significant resources that provide a basis for future discovery in the study of type 1 diabetes genetics.

## Introduction

The importance of studying diverse groups of individuals and the need for increased sample sizes to answer specific disease questions have led to the conduct of international trials and consortia in the past decade [1–16]. While some publications regarding the challenges faced in conducting an international study are available, there is the need for more published information to define potential issues and solutions.

To pool data obtained from such efforts, it is critical to standardize the collection procedures across all sites worldwide. This may prove to be a formidable task, with a variety of issues not fully appreciated from the outset of such a project. The addition of sites worldwide adds complexity and considerable time to the planning and implementation processes.

The Type 1 Diabetes Genetics Consortium (T1DGC) is an international project sponsored by the National Institute for Diabetes and Digestive and Kidney Diseases (NIDDK) and the Juvenile Diabetes Research Foundation (JDRF) whose primary aims are to: (a) discover genes that modify the risk of type 1 diabetes; and (b) expand upon the existing genetic resources for type 1 diabetes. The initial Consortium goal was to collect 2500 affected sibling pair (ASP) families throughout the world. These families would provide medical history information as well as samples for immortalized cell lines, DNA, plasma, and serum. All samples eventually will be deposited in the NIDDK Central Repositories and made available to the scientific community.

## Methods

### Study organization

**Figure 1:**
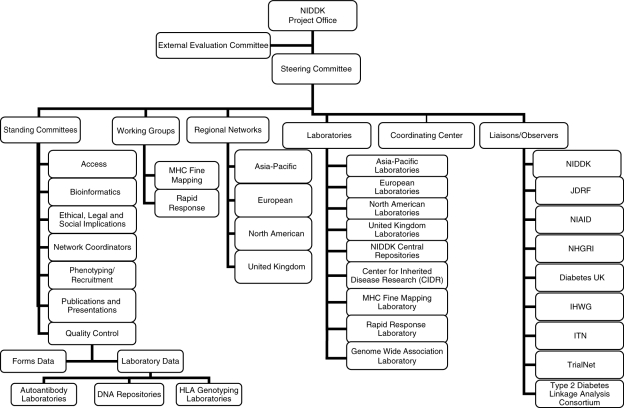
T1DGC organization chart.

Defining the study organization is an important first step in developing the necessary infrastructure to undertake such a project. The T1DGC has its Project Office at NIDDK and includes a Steering Committee, an External Evaluation Committee (EEC), Network Centers, Network Laboratories, Standing Committees and a Coordinating Center as well as liaisons and program observers from various National Institutes of Health (NIH) agencies and studies. Figure 1 illustrates the size and complexity of this project.

#### Steering Committee

The T1DGC Steering Committee was responsible for the overall T1DGC study. Steering Committee investigators participated in the design and execution of the project and collectively approved decisions for the Coordinating Center to execute. Members included representatives from each regional Network, the Coordinating Center, and program staff from the sponsoring organizations. Decisions were made by a majority vote of a quorum of the committee members. The Steering Committee met by conference call once a month and in face-to-face meetings twice per year.

#### External Evaluation Committee

NIDDK established an EEC that was responsible for ongoing evaluation of the study design and monitoring the progress of the T1DGC. EEC members included investigators with relevant scientific expertise, but who were not the members of the Consortium.

#### Network Centers

To facilitate participant recruitment, the Consortium was organized into four regional Networks: Asia-Pacific, European, North American, and United Kingdom. The Asia-Pacific Network Center was located at the Walter and Eliza Hall Institute of Medical Research in Melbourne, Australia, and had 20 clinics. The European Network Center was located at the Hagedorn Research Institute (formerly Steno Diabetes Center) in Gentofte, Denmark, with 84 clinics. The North American Network Center was located at Benaroya Research Institute in Seattle, WA, USA, and had 62 clinics. The United Kingdom Network Center was located at the University of Cambridge and included 48 clinics. A total of 214 clinics in 34 countries participated in recruitment for T1DGC.

Each network was responsible for coordinating and monitoring all the clinic activities within the region. Each of the four networks established a network infrastructure, developing the Network Center and regional organizations through contacts with investigators and clinicians with ASP families to contribute to the collection. A Network Coordinator was appointed for each region. Each network was given flexibility to develop its region as deemed necessary for the overall success of the Consortium.

To identify participating clinics, network meetings were held to outline the T1DGC collection requirements (*i.e.,* data and samples required for inclusion) and to determine investigator interest and feasibility of participation. Following such meetings, the regional Network Coordinator would obtain detailed clinic information, such as the estimated number of available families, staff contacts, and local or national issues that might prevent participation in the Consortium.

Each regional Network Center was responsible for coordinating and monitoring study activities within the region. Network Centers worked with investigators at participating clinics to prepare materials for submission to Institutional Review Boards (IRBs) and Ethical Committees (ECs). Network Centers performed all data entry and maintained continuous interaction with network clinics and laboratories as well as the Coordinating Center.

#### Laboratories

Each of the four regional networks established three types of laboratories to perform activities integral to meeting the study goals: a DNA Repository to establish cell lines and extract DNA for genotyping projects; an Autoantibody and Storage Laboratory for measurement of autoantibodies and temporary storage of serum and plasma samples; and a Human Leukocyte Antigen (HLA) Genotyping Laboratory for HLA characterization. A quality assurance (QA) plan was established and implemented; assays were standardized across each type of laboratory. Internal quality control (QC) data or any existing comparisons between laboratories were submitted to and reviewed at the Coordinating Center. All of the T1DGC laboratories participated in annual comparisons and/or QC exercises.

Other laboratories were selected for specific genotyping projects. These included the Center for Inherited Disease Research (CIDR) (Johns Hopkins University, Baltimore, MD, USA; genotyping for linkage), The Wellcome Trust Sanger Institute (Hinxton, UK; fine mapping of the major histocompatibility complex (MHC) region), and The Broad Institute Center for Genotyping and Analysis (Cambridge, MA, USA; evaluation of candidate genes for type 1 diabetes). Data from these projects were sent to the Coordinating Center for additional QC checks prior to data distribution and analyses.

#### Coordinating Center

The T1DGC Coordinating Center (Division of Public Health Sciences, Wake Forest University Health Sciences, Winston-Salem, NC, USA) monitored and supported data collection activities within the four Network Centers. Since the regional Network Centers were charged with coordinating and monitoring all clinic activities within the region, the Coordinating Center interacted only with the Network Centers and not with individual clinics in a region.

The Coordinating Center established and maintained QA standards for all activities of the study and worked with the Network Centers and laboratories to implement decisions made by the T1DGC Steering Committee. In addition, the Coordinating Center was responsible for fiscal administration of the project.

**Figure 2:**
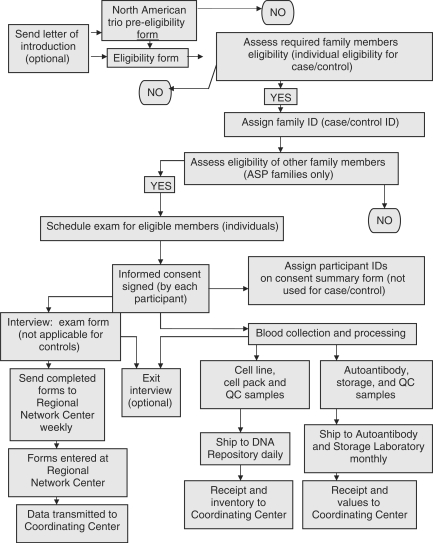
T1DGC data and sample collection flow.

**Figure 3:**
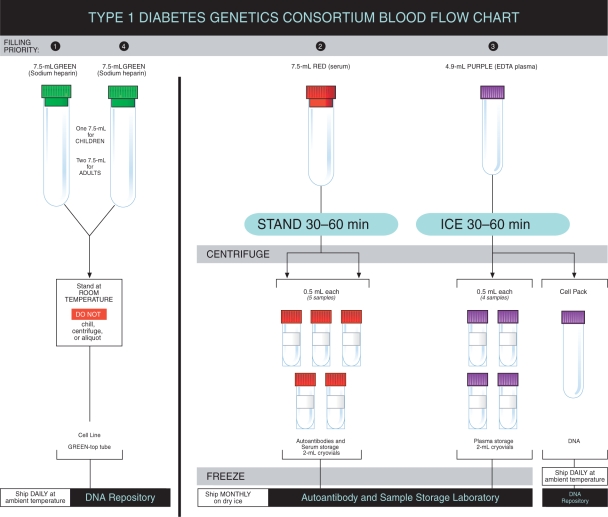
T1DGC blood collection chart.

Three specialized teams were established, each focusing on specific aspects of the study (*i.e.,* Operations, Systems, and Statistics). The Operations Team, in collaboration with regional representatives, developed all study materials, including: a template for informed consent; the protocol; a manual of operations; and data collection forms for ASP, trio, and case–control collections. Revisions to each of these documents were implemented as needed. Included in the manual of operations were figures to provide visual references for key aspects of the data and sample collection (Figures 2 and 3). The Systems Team was responsible for data flow, architecture, and security. This team developed and finalized two study websites (T1DGC public site (www.t1dgc.org) and an internal T1DGC data entry site for certified Network Center and laboratory personnel) as well as other fully web-based applications, including a specimen tracking system and a HLA genotyping laboratory system. The Statistics Team was responsible for data management, QA/QC, data set creation and distribution, and initial analyses.

#### Standing Committees and Working Groups

Ten standing committees were established to implement Consortium activities and provide opportunities for T1DGC members to participate. Each committee included representatives from the four regional networks, the Coordinating Center, and the sponsoring organizations. T1DGC committees included: Access; Bioinformatics; Ethical, Legal, and Social Implications (ELSI); Network Coordinators; Phenotyping/Recruitment (including eligibility review and approval); Publications and Presentations; and four QC Committees (Autoantibody, DNA Repository, HLA Genotyping, and Forms Data). Monthly calls with each of the QC Committees were used to review QC reports and to discuss laboratory-specific issues. Other committees scheduled calls as required to deal with specific study issues. Face-to-face meetings of all T1DGC committees were held annually.

In addition to the Standing Committees, T1DGC established two Working Groups (MHC and Rapid Response) to analyze data associated with two genotyping projects. Each group comprised experts in the specific regions that were genotyped.

### Training, certification, and pilot studies

Cultural and language differences made study-wide, central training sessions difficult. T1DGC used a ‘train the trainer’ model where Network Center staff members were trained at the Network Center by Coordinating Center staff. The Network Coordinator, in turn, was responsible for subsequent training of clinic staff, either centrally or individually. This model enabled networks to initiate data collection on a staggered timetable.

Following training, each participating clinic was required to conduct a pilot study before initiating T1DGC data collection. Data were reviewed by a Coordinating Center Project Manager who certified or provided final approval for the clinic to begin T1DGC participant recruitment. All data collection forms were data entered at the Network Center by staff trained and certified in the data entry system.

### Quality control

The Coordinating Center established QA procedures and QC metrics for all Consortium activities. These activities included the data collection forms entry [17], sample assays for the Network Laboratories [18–20], and any genotyping performed on the samples [21,22]. T1DGC samples were deposited at the NIDDK Central Repositories and the Coordinating Center worked with NIH staff to assure that samples received were of high quality.

## Results and lessons learned

### Organization

The T1DGC Steering Committee was responsible for the overall T1DGC study and members actively participated in the design and execution of the project. From the outset, T1DGC decided that a distributed organization of regional networks was necessary to complete a worldwide recruitment of 2500 ASP families. Four regional Networks (in Asia-Pacific, Europe, North America, and the United Kingdom) were organized, with the aim to ensure standardized collection procedures across all sites worldwide. This proved to be a formidable task, with a variety of issues not fully identified from the outset of such a project.

An important component of the success of this consortium was the development of the T1DGC Consortium Agreement (Appendix 1) that incentivized investigator participation. This agreement clearly defined the activities of the Consortium and member rights and responsibilities. The agreement acknowledged contributing investigators and explicitly respected their research prerogatives. It also outlined a timeline for making T1DGC resources available to contributing investigators, to T1DGC members, and to the broader research community.

### Recruitment

To facilitate worldwide recruitment, each network was given flexibility to develop its own Network Center and regional organization to meet the overall participant recruitment goals. While this resulted in very different approaches across the four networks, it led to the overall success of Consortium recruitment, as each network could deal with the unique social, cultural, ethical, and legal issues of different countries. This flexible approach proved to be an effective and successful strategy.

Network meetings were a key factor in facilitating interaction among participating investigators within networks. For example, the initial network meetings outlined the T1DGC collection requirements and determined investigator interest and feasibility of participating. Subsequent network meetings, generally on an annual basis, provided updates on the status of recruitment, activities of the Consortium, and new developments in type 1 diabetes genetics. At some subsequent network meetings, additional training was provided for areas of the study that required more emphasis and training for new aspects of the study was conducted.

The centralization of some activities combined with the delegation of other activities contributed to the smooth running of the Consortium and the success of recruitment. Initially, T1DGC collected only ASP families. Later, on the recommendation of the Steering Committee and the approval of the EEC, T1DGC included the recruitment of trios (father, mother, and a child with type 1 diabetes), as well as cases (with type 1 diabetes) and controls (no history of type 1 diabetes) from populations with a low prevalence of the disease. In Asia-Pacific, these included individuals from India, Thailand, Malaysia, Philippines, and Singapore. In Europe, Cameroon was included to provide trios, cases, and controls. In North America, Mexican-American, and African-American individuals were included. Table 1 provides a summary of the T1DGC recruitment and basic demographics for eligible participants as of July 4, 2009. Recruitment and data cleaning are ongoing.

**Table 1:** Demographics of completed affected sibling pair families, trios, cases, and controls by network, T1DGC, July 4, 2009

	Asia-Pacific	European	North American	United Kingdom	Overall
**Affected sibling pair families**					
Number completed families	324	1215	1153	163	2855
*Gender (percent)*					
Male	46.6	48.6	49.3	45.2	48.4
Female	53.4	51.4	50.7	54.8	51.6
*Race (percent)*					
American Indian/Alaskan Native	0.0	0.0	0.2	0.0	0.1
Asian	6.7	0.0	0.7	1.3	1.2
Native Hawaiian or other Pacific Islander	1.3	0.0	0.2	0.0	0.2
Black or African American	1.6	0.2	2.2	0.7	1.2
White or Caucasian	90.3	99.8	96.7	97.9	97.3
*For affected participants (mean ± SD^a^)*					
Age at ascertainment (proband)	21.7 ± 12.4	25.6 ± 13.3	21.3 ± 12.9	16.7 ± 6.4	22.9 ± 13.0
Age at diagnosis (proband)	8.0 ± 6.3	9.7 ± 7.1	7.6 ± 5.7	6.2 ± 4.3	8.5 ± 6.4
Age at ascertainment (affected siblings)	20.0 ± 12.2	24.1 ± 13.2	19.7 ± 13.0	14.2 ± 5.8	21.3 ± 13.0
Age at diagnosis (affected siblings)	12.8 ± 8.4	14.8 ± 9.0	11.5 ± 7.9	8.8 ± 4.4	12.9 ± 8.5
**Trio families**					
Number completed families	269	11	192	N/A^b^	
*Gender (percent)*					
Male	48.5	60.0	47.5	N/A	48.2
Female	51.5	40.0	52.5	N/A	51.8
*Race (percent)*					
American Indian/Alaskan Native	0.0	0.0	0.0	N/A	0.0
Asian	100.0	0.0	0.0	N/A	57.5
Native Hawaiian or other Pacific Islander	0.0	0.0	0.0	N/A	0.0
Black or African American	0.0	100	48.0	N/A	21.2
White or Caucasian	0.0	0.0	52.0	N/A	21.3
*For affected participant (mean ± SD)*					
Age at ascertainment (proband)	16.5 ± 7.4	14.7 ± 4.8	11.1 ± 4.6	N/A	14.3 ± 6.9
Age at diagnosis (proband)	10.2 ± 5.4	11.3 ± 4.2	7.2 ± 4.0	N/A	9.0 ± 5.0

**Cases**					
Number completed	4	0	390	N/A	394
*Gender (percent)*					
Male	100.0	0.0	46.0	N/A	46.5
Female	0.0	0.0	54.0	N/A	53.5
*Race (percent)*					
American Indian/Alaskan Native	0.0	0.0	0.0	N/A	0.0
Asian	100.0	0.0	0.0	N/A	1.0
Native Hawaiian or other Pacific Islander	0.0	0.0	0.0	N/A	0.0
Black or African American	0.0	0.0	77.7	N/A	76.9
White or Caucasian	0.0	0.0	22.3	N/A	22.1
Age at ascertainment (mean ± SD)	21.8 ± 3.9	0.0	14.8 ± 7.7	N/A	14.9 ± 7.7
Age at diagnosis (mean ± SD)	14.2 ± 2.9	0.0	8.9 ± 5.4	N/A	8.9 ± 5.5

**Controls**					
Number completed	2	0	527	N/A	529
*Gender*					
Male	100.0	0.0	23.6	N/A	23.9
Female	0.0	0.0	76.4	N/A	76.1
*Race*					
American Indian/Alaskan Native	0.0	0.0	0.0	N/A	0.0
Asian	100.0	0.0	0.0	N/A	0.4
Native Hawaiian or other Pacific Islander	0.0	0.0	0.0	N/A	0.0
Black or African American	0.0	0.0	81.0	N/A	80.7
White or Caucasian	0.0	0.0	19.0	N/A	18.9
Age at ascertainment (mean ± SD)	23.0 ± 1.4	0.0	32.8 ± 12.8	N/A	32.8 ± 12.8

a SD – standard deviation. ^b^N/A – not applicable.

The protocol, manual of operations, and data collection forms were developed centrally at the Coordinating Center, with input from network representatives. All study documents were made available on the T1DGC website (www.t1dgc.org).

T1DGC standardized supplies and services worldwide by establishing central billing accounts and using vendors that would permit clinics to order from a common catalog of supplies. Central billing accounts were created for: blood collection supplies to be used in the clinics (Sarstedt, Inc.); fetal bovine serum to be used in establishing cell lines in the Network DNA Repositories (Invitrogen, Inc.); and couriers to ship specimens from the clinics to laboratories and from DNA Repositories to genotyping facilities (Federal Express and World Courier). Locating vendors and establishing the master accounts took considerable time and effort, so the decision to pursue this type of arrangement should be made as early as possible in the planning process to avoid delays in data collection. For instance, when T1DGC realized that there could not be a single worldwide courier for shipping samples, an account with Federal Express was used for shipments within North America and another account with World Courier was used for shipments in the Asia-Pacific and European Networks. In the United Kingdom, no courier master account was required as the postal system was used for shipping cell line samples to Cambridge and a local van courier for frozen shipments to the laboratory in Bristol.

### Regulatory issues

Every institution engaged in human subjects research supported or conducted by the US Department of Health and Human Services (DHHS) must obtain an assurance of compliance approved by the Office for Human Research Protections (OHRP). Some international institutions did not have an active Federal Wide Assurance (FWA) number and this was a primary cause of delayed recruitment in clinics. Some networks overcame this issue by using an Unaffiliated Investigator Agreement, where one institution agreed to serve as an umbrella for other collection sites. Clinic sites also were encouraged to register their IRB and apply for an FWA number online at the OHRP website. As with other studies, obtaining IRB or EC approval was the major source of delay in initiating recruitment, as each IRB or EC had its own set of requirements.

The T1DGC ELSI Committee dealt with the large number of issues related to informed consent [23]. This group finalized a set of templates (self consent, parental consent, teenage assent, and child assent) that was agreed to by all networks. Templates could be modified to comply with local IRB or EC requirements, as long as a defined set of specific elements required for the T1DGC collection were included in the final approved version.

Dealing with informed consent language is a time-consuming task in any study, but was particularly so in T1DGC, given the diverse requirements necessary to satisfy hundreds of IRBs or ECs. Particular to a genetics study, T1DGC had to be sensitive to specific cultural issues about the collection of genetic material and to reassure investigators from countries who felt that genetics collection was primarily an exploitive activity. T1DGC added language to the consent templates to specifically state that the Consortium would not claim any intellectual property rights, sell the DNA, or develop any commercial products.

In North America, several US IRBs required the T1DGC to apply for and obtain a Certificate of Confidentiality. To ensure compliance with the Health and Insurance Portability and Accountability Act (HIPAA), the Coordinating Center developed and executed Data Use Agreements for transfer of data between each of the Network Centers and the Coordinating Center.

### Study communication

In general, T1DGC committees had monthly conference calls throughout the study. Email was the primary means of communication between the Coordinating Center and the Network Centers, especially in the intervals between conference calls. Face-to-face meetings of committee members occurred annually.

T1DGC greatly benefited from web-based communications. There were two study websites: a public site with login access for Consortium Members and an internal data entry website used for input and access to all study data that was accessible only to specified study personnel. Since data were available to all Network Coordinators, real-time monitoring of recruitment was possible and was used as an incentive to spur recruitment efforts. Network laboratories used the data entry site to report their results to the Coordinating Center. The T1DGC also developed a web-based application for the HLA Genotyping Laboratories to report their results [20].

The T1DGC website (www.t1dgc.org) was used to communicate with the general T1DGC membership and the public. Specific pages of the website were used for communications with different T1DGC committees and working groups, including the Steering Committee. The Consortium Agreement, access policies, and copies of data collection forms were all available on the T1DGC website.

### Quality control

The T1DGC Steering Committee appreciated that having standardized assays and/or methods across network laboratories would be critical for the success of the study. The T1DGC study data are primarily of two types: medical history information recorded on data collection forms and laboratory results. Since there were three types of laboratories within each network, the central QC Committee was comprised of four subcommittees: (1) Forms Data; (2) DNA Repositories; (3) Autoantibody Laboratories; and (4) HLA Genotyping Laboratories.

The QC Committee and the Coordinating Center developed QA procedures, including a central manual, for use in all networks. To implement QA, the QC Committee reviewed internal QC data or any existing comparison data among the network laboratories and also conducted annual comparisons of laboratories worldwide. This ensured consistency and allowed the study to monitor for assay drift.

The QC Committee conducted site visits to each regional Network Center and Network Laboratory to monitor adherence to the protocol under normal operating conditions. Site visits also were used to identify and resolve any data collection issues at individual clinics and/or any questions about sample shipments, handling, and analysis procedures at the laboratories. It proved challenging, but possible, to train and to impose rigorous QA procedures across networks. Again, insistence on uniform standards, with flexibility on specific details, was critical to implementing the established QA standards.

### Access

One of the main goals of the T1DGC was to share its data, samples, and resources with the broader research community. This goal was prominently stated in the Consortium Agreement and was implemented by the Access Committee. The T1DGC access policy is available on the website (www.t1dgc.org) and included as Appendix 2. Importantly, there is a prominent banner on the website that highlights data and sample availability. There is also a list of all investigators who have been provided access to T1DGC resources (samples and/or data).

The T1DGC is depositing samples and data in all three NIDDK Central Repositories (Biosample, Genetics, and Data). The Central Repositories were established to expand the utility of NIDDK-supported studies by allowing the research community to continue to access these materials beyond the end of the study.

T1DGC conducted training workshops for HLA genotyping and for bioinformatics in a conscious effort to export technology, providing hands-on opportunities for T1DGC members. Since real T1DGC samples and data were used, these workshops also highlighted the availability of the resources.

### Genotyping

All genotyping of T1DGC samples was performed centrally, although different laboratories were used for different aspects of the study. The Coordinating Center worked with the Network DNA Repositories to ship samples to the selected facility. Genotype data were returned to the Coordinating Center, where the Statistics Team was responsible for performing additional QC checks and initial analyses. The results of all analyses undertaken by T1DGC have been made available to the T1DGC membership and announced on the T1DGC website.

The initial T1DGC activity was a joint analysis of three historical genome-scan data sets (UK, US, and Scandinavia), combined with data from 254 T1DGC ASP families that had been genotyped by CIDR. These results were published as an Original Article in *Diabetes* [24]. This was followed by genotyping all T1DGC-collected ASP families, with genotyping performed by CIDR and the results published in *Diabetes* [25].

The T1DGC subsequently genotyped ∼10,000 samples at The Wellcome Trust Sanger Institute to generate a data set of single nucleotide polymorphisms (SNPs) and microsatellites within the 4 Mb classical MHC region. This information was combined with HLA class I and class II genotyping performed in the T1DGC HLA Genotyping Laboratories. This comprehensive, unique data resource was made available to multiple analytic working groups. Their results are presented in a supplement of *Diabetes, Obesity and Metabolism* [26].

The same ∼10,000 DNA samples have been used to investigate previously reported candidate genes for type 1 diabetes, to confirm the most highly associated SNPs reported by the Wellcome Trust Case Control Consortium (WTCCC) [27], to study recently reported genes that contribute to risk of type 2 diabetes and are implicated in β-cell function, and to interrogate recently reported genes from genome-wide association studies (GWAS) of other autoimmune diseases. Genotyping for these projects was performed at The Broad Institute and the results are presented in a supplement of *Genes and Immunity* [28].

The T1DGC has completed Stage 1 of a GWAS, using ∼500,000 SNPs in 4000 cases from the JDRF/Wellcome Trust British case collection and 2500 controls from the British 1958 Birth Cohort (B58C). These results were combined with existing data from the WTCCC study of type 1 diabetes [27] and from the Genetics of Kidneys in Diabetes (GoKinD) Study [29]. Genotyping was by contract to Illumina and Professor David Clayton (University of Cambridge, UK) led the analysis. The results have been published in *Nature Genetics* [30]. Follow-up studies to the GWAS Stage 1 currently are underway and are utilizing T1DGC samples.

### Limitations

The original T1DGC research plan for a linkage study guided the decision to collect ASP families. From the outset, the Steering Committee recognized that the rapid development of genotyping technologies would make it necessary to anticipate modifications in the research design. Indeed, one rationale for establishing the EEC was to be able to obtain peer review of any proposed design changes and solicit agreement from the sponsoring organizations for implementation of these changes.

While the T1DGC infrastructure was established to collect ASPs, flexibility to accommodate design changes was built in, to the extent possible. This was especially true with respect to the language of study materials provided to the IRBs. The approval for recruitment of trios and case–control collections added complexity to the study. New data collection forms had to be designed and additional training had to be initiated. However, most IRBs considered the addition of new cohorts as a modification of the approved protocol and provided expedited review.

Although the T1DGC established four regional networks, the designation of networks was arbitrary (to some extent) and resulted from early participation of investigators from those regions in the planning of the project. Unfortunately, T1DGC could not accommodate investigators in regions or countries who wanted to have their own network. Each regional Network in turn designated a set of network laboratories. T1DGC insisted on rigorous QA procedures and was able to achieve and maintain quality performance across the different network laboratories. Investigators from some countries declined to participate in T1DGC, arguing that they had laboratories and technologies in their own countries and refusing to send samples to a central location. Finally, it was a condition for funding that samples collected for T1DGC must be exported and deposited in the NIDDK Central Repositories. These conditions prevented certain countries (*e.g.,* China, Japan and Korea) from participating. Recruitment for a future worldwide genetics project might take these limitations into account and begin with pilot collections in several nations to increase confidence and accommodate differences.

As a genetics study (and not, for example, a clinical trial), T1DGC planned no sustained contact with the participants. In some networks, no re-contact was explicitly stated in the consent form. As data on T1DGC participants accumulates, it would have been useful to be able to re-contact participants for collection of additional samples to carry out functional studies.

## Conclusions and recommendations

The T1DGC is a NIDDK- and JDRF-sponsored project whose primary aims are to: (a) discover genes that modify risk of type 1 diabetes and (b) expand upon existing genetic resources for type 1 diabetes research. T1DGC set an ambitious recruitment target of 2500 ASP families worldwide and established the organization and infrastructure that completed this recruitment and also collected additional trios, cases, and controls.

T1DGC’s four regional Networks (Asia-Pacific, European, North American, and United Kingdom) were responsible for coordinating recruitment activities within each region, but were given the flexibility to develop that region as deemed necessary for the overall success of the Consortium. This flexibility meant that each network could deal sensitively with the particular social, cultural, ethical, and legal issues of their different countries.

Standardized data collection was an overarching goal of the T1DGC – even with worldwide recruitment, sample handling, and analysis. The T1DGC Coordinating Center monitored and supported the activities within the four Network Centers. The protocol, manual of operations, and forms were developed centrally at the Coordinating Center, with input from network representatives. The Coordinating Center developed QA procedures and implemented quality monitoring for all networks, including the network laboratories. Good communication and expedient problem solving are key requirements for success.

The T1DGC data and sample collection includes ASP families, trios, cases, and controls. Results of genetic analyses have identified many novel new regions that affect susceptibility to type 1 diabetes [24–30]. T1DGC data and samples are accessible to the research community and should prove to be particularly rich resources well into the future.

## Appendix 1

### Type 1 Diabetes Genetics Consortium Agreement

#### Background

The international Type 1 Diabetes Genetics Consortium (T1DGC) is a collaborative group formed to facilitate the genetic analysis of type 1 diabetes (T1D) via the sharing of reagents, methods, strategies, samples, knowledge, and data at all levels of the research effort, from individual groups to existing and future collaborative networks. The T1DGC will help both researchers and funding agencies monitor progress, and formulate, plan, and assess the most informative and cost-effective strategies. It will help motivate researchers, peer reviewers, and lay people to push forward the identification of genes and mechanisms in T1D. It will encourage sample and data sharing while maintaining the environment for new initiatives, both small and large, across the wide spectrum of approaches and technologies required in genetic analysis of complex diseases.

This initiative is undertaken in order to help increase consensus in the field, to provide an opportunity to collate data from a variety of studies for combined analyses and to increase clarity for future initiatives. A major role for the T1DGC will be to act as a repository for data and samples, to support scientists worldwide and to encourage new ideas for research in Type 1 diabetes.

#### Activities of the Consortium

T1DGC has the following activities:

1. The Consortium will provide an integrated map and new analysis of the combined data set of affected sibling pair (ASP) families (*n* ∼ 1200). The combined data set includes genome-scan data from a combined US–UK collection (*AJHG* 2001, 69:820-830), and from a Scandinavian collection (*AJHG* 2001, 69:1301-1313). This analysis is ongoing. The data will be made available to all investigators on request when the manuscript has been accepted.
2. The Consortium will transmit samples from approximately 600 ASP families already collected by various investigators to the Center for Inherited Disease Research (CIDR) for whole-genome scan analysis. This submission has already occurred.
3. The Consortium will organize and collect an additional 2500 ASP families throughout the world. In order to conduct this recruitment, the Consortium has been organized into regional Networks. These families will provide DNA, plasma and serum samples, and phenotypic and medical history information. Family members will also be asked to allow immortalized cell lines to be made. A whole genome-scan analysis will be conducted on the DNA from recruited families. All samples (DNA, plasma, serum, cell lines) will eventually be deposited in a central NIDDK repository and be made available to the scientific community.
4. The Consortium has received funding to identify genes under the five most promising linkage peaks identified by the analysis. The Steering Committee will develop specific procedures for this identification.
5. Such procedures in the future could include association studies and genetic analyses of diverse ethnic groups, using trios, cases, and controls.

#### Steering Committee of T1DGC

The T1DGC will be guided by a Steering Committee (SC), the membership of which is as follows:

**Table:**

Member	Institution	Location	Country
Beena Akolkar	NIDDK	Bethesda, MD	USA
Pat Concannon	University of Virginia	Charlottesville, VA	USA
Henry Erlich	Roche Molecular Systems/CHORI	Oakland, CA	USA
Cecile Julier	Centre National de Genotypage	Paris	France
Grant Morahan	Western Australian Institute for Medical Research	Perth	Australia
Jorn Nerup	Hagedorn Research Institute	Gentofte	Denmark
Flemming Pociot	Hagedorn Research Institute	Gentofte	Denmark
Stephen Rich (Chair)	University of Virginia	Charlottesville, VA	USA
John Todd	University of Cambridge	Cambridge	UK

Under the terms of funding, the NIDDK Program Officer is a full member of the SC.

Decisions are made by the Steering Committee by majority vote of a quorum of its membership. SC Voting Members are: Akolkar, Concannon, Erlich, Julier, Morahan, Nerup, Pociot, Rich, and Todd. Seven SC members constitute a quorum empowered to conduct T1DGC business. A simple majority vote (4/7 or 5/8 or 5/9) is considered binding.

The SC has established several committees to help with the functioning of the Consortium. These committees are: Access; Bioinformatics; Ethical, Legal, and Social Implications (ELSI); Molecular Technology; Phenotyping/Recruitment; Publications and Presentations; and Quality Control.

The Coordinating Center for T1DGC has been established at Wake Forest University.

The T1DGC has funding from the NIDDK (NIH) and the Juvenile Diabetes Research Foundation (JDRF) to initiate a worldwide collection of affected sibling pair (and other pedigree types) families for on-going linkage studies and future association studies.

#### Network Organization

The T1DGC framework will support and foster Regional Networks. The Network PIs, working with physicians in local clinics, will be responsible for recruiting T1D subjects and families and for upholding local ethical conditions, informed consent, and compliance.

The Regional Networks and PIs are:

**Table:**

Network	PI	Institution	Country
Asia-Pacific	Peter Colman	Walter & Eliza Hall Institute	Australia
	Grant Morahan		
European	Jorn Nerup	Hagedorn Research Institute	Denmark
	Flemming Pociot	Hagedorn Research Institute	Denmark
North American	Carla Greenbaum	Benaroya Research Institute	USA
United Kingdom	John Todd	University of Cambridge	UK

Network investigators will be subject to national and regional laws and regulations in force. This includes appropriate ethical review and approval. Study subjects will have the right to request that their samples and information be destroyed at any time in the future. The details of the procedure for this request will be determined by Network policy.

#### Consortium Member Rights and Responsibilities

Investigators who choose to participate in the Consortium agree to abide by the following principles:

1. Each participating group will indicate their willingness to participate in the consortium effort and to abide by the principles outlined in this Consortium Agreement by providing a signed copy of this Consortium Agreement. By signing this Agreement, the investigator will receive Member access to the T1DGC website (http://www.t1dgc.org).
2. There are several ways to participate in the Consortium. These include:
  – participating in the recruitment of new collections for and on behalf of the Consortium, using Consortium resources
  – providing genotyping or other research resources to the Consortium
  – participating in the activities of Consortium committees
  – participating in the analysis of aggregate Consortium data
3. Some investigators have provided previously collected samples for submission to CIDR, as indicated in activity *2*, above. In this case, the contributing investigator has agreed:
  – to be eligible to receive access to the data derived from the analysis of the samples they contributed to the Consortium, including HLA, *INS*, and *CTLA4* typing and genome scan data;
  – to retain the right to analyze and publish their own data, with no non-scientific restrictions on timing or content;
  – to retain any intellectual property rights deriving from their separate analysis and/or publication of their own data;
  – to participate in any joint analyses conducted by the Consortium, and to keep any interim results of such analyses confidential;
  – to be recognized in publications to be co-authored by ‘The Type 1 Diabetes Genetics Consortium’.
4. An investigator may choose to participate in the Consortium by helping to recruit families for the Consortium collection indicated as activity *3*, above. A group that contributes to the recruitment of new collections for and on behalf of the Consortium agrees to the following:
  – to participate in the discussions leading to the establishment of Consortium standards for collection, and to abide by those standards;
  – to obtain signed informed consent from volunteers according to high ethical and legal standards;
  – to provide the Consortium-determined quantities of blood, serum, plasma, and other samples, and to send these biological samples to Consortium-designated laboratories;
  – to obtain phenotype and medical history information from volunteers, and to report this information to a Consortium-designated Center;
  – to inform the Consortium if there are any ancillary studies being conducted while recruiting for the Consortium. An investigator agrees to provide the set of data and samples required by the Consortium.
  – Laboratory results may be returned to the investigator, depending on Network policy. It is up to the discretion of the investigator to share this information with the subject, according to individual data collection site requirements. It is the responsibility of the Network to develop any statements about the interpretation of data, and to request that this statement be shared with participants.
  – In the case where site requirements mean no laboratory results will be returned to the subject, and the subject makes an explicit request for this information, a second sample will be sent to another laboratory designated by the subject. The subject will pay for this testing and results.
  – Investigators must agree to destroy any samples and information when subjects have requested withdrawal from the study. Investigators must notify their regional Network of any request for destruction of samples and information. The details of the procedure for this request will be determined by Network policy.
5. A group that contributes to recruitment for and on behalf of the Consortium has the following rights:
  – The Consortium is committed to providing data to contributors. It is the responsibility of the contributing investigator to obtain the appropriate ethical and privacy board review and approvals for receiving coded data and materials from the Consortium. Contributors may be required to provide these assurances prior to data and/or materials release.
  – Contributors will receive access to data derived from the analysis of the samples they contributed to the Consortium, including HLA, *INS*, and *CTLA4* typing and genome scan data. For NIH purposes, the T1DGC Coordinating Center (CC) must track who requests data and where it goes. Therefore, all investigators have to make written requests for data; email is acceptable. Contributors can request immediate access to the genome scan data for their own analysis, and the CC will provide that data as soon as the data are received.
  – Contributing investigators have the right to receive DNA and/or immortalized cell line aliquots of samples they contributed to the Consortium.
  – Contributing investigators retain the right to analyze and publish their own data, with no non-scientific restrictions on timing or content.
  – Contributors retain any intellectual property rights deriving from their separate analysis of and/or publication of their own data.
  – Contributors have the right to participate in any joint analyses including the data from their samples conducted by the Consortium, and agree to keep any interim results of such analyses confidential.
  – Contributors agree to acknowledge the Consortium in publications and presentations, according to the policies determined by the Consortium Publications and Presentations Committee.
  – Contributors will be recognized in publications to be co-authored by ‘The Type 1 Diabetes Genetics Consortium’.
  – Contributors will be eligible to apply for access to Consortium resources for additional analyses, according to policies and timetables established by the Consortium Steering Committee.

#### Consortium Responsibilities

1) The Consortium agrees to the following:
  – to establish mechanisms for interested groups to participate in the activities of the Consortium;
  – on request, to return the results of analysis of samples provided by contributing investigators;
  – on request, to provide an aliquot of DNA and/or immortalized cell lines made from samples provided by contributors;
  – on request, to notify the NIDDK Central Repository of any request to destroy contributed samples and information;
  – to recognize the right of contributors to analyze and publish their own data, with no non-scientific restrictions on timing or content;
  – to establish mechanisms for contributors to participate in any joint analyses including the data from their samples conducted by the Consortium;
  – to recognize the contributing groups and individuals in publications to be co-authored by ‘The Type 1 Diabetes Genetics Consortium’.
2) The Consortium will transmit collected DNA samples to the Center for Inherited Disease Research (CIDR) for whole-genome scan analysis. Because the samples will be assembled from a worldwide recruitment, the Consortium expects samples to be ready in a staggered schedule. Approximately 800 families will be provided in each of three submissions to CIDR. Each Network will have samples in each submission to CIDR.

An anticipated schedule for submissions to CIDR, receipt of data from CIDR and data releases is according to the following approximate timetable:

**Table:**

Submission to CIDR	Data received from CIDR	Expected release of aggregate data analysis*
January 2003	October 2003	+6 months
December 2004	August 2005	+6 months
December 2005	August 2006	+6 months
December 2006	August 2007	+6 months

* The Consortium expects to release the results of the aggregate analysis incorporating the samples from each submission according to the approximate schedule above.

On request from contributing investigators, the Consortium will provide whole-genome scan data on the contributed samples as soon as it is received.

The Consortium will undertake quality control analysis of the data and will also begin linkage analysis on the aggregate data. Linkage analysis will be done only on the aggregate data set, and according to policies determined by the Steering Committee. Contributors have the right to participate in any joint analyses that includes data from their samples that are conducted by the Consortium. Contributors agree to keep any interim results of such analyses confidential.

The Consortium will release the genotypic data to Consortium Members from each submission 6 months after receipt of the data from CIDR.

At the conclusion of the study, the Consortium will create a database for the NIDDK Central Repository, according to the requirements of the Repository. Access to Consortium data will then be governed by policies determined by the NIDDK Central Repository.

The Consortium has no expectation that patentable information or material will result from the combined or aggregate Consortium database, and the Consortium will not assert or claim any such intellectual property (IP) rights. It may be possible for individual investigators who follow up the linkage results to generate patentable information or material, but they will need to decide themselves whether to claim any such potential intellectual property.

3) The Consortium agrees to provide resources for genetic analyses to the scientific community. The Consortium has received funding to identify genes under the five most promising linkage peaks identified by the analysis. The Steering Committee will develop specific procedures for this identification. For additional research in the genetics of type 1 diabetes, the following working model is being developed:
  - Investigators interested in identifying genes may apply for access to Consortium samples.
  - Applications would be reviewed by an Initial Review Group (IRG) acting according to policies drawn up by the Access Committee, and ratified by the Steering Committee.
  - The IRG would include *ad hoc* reviewers, outside scientists (with expertise not in diabetes), and representatives of the funding agencies.
  - Multiple applications may result in recommendation that collaborations be implemented. This would ensure that high-cost, labor-intensive positional cloning would be done with minimum overlap among labs.
  - IP issues would be negotiated between or among investigators working on a particular region.
  - When publication is in press, the data would be provided to the Consortium. The Consortium would make the data available after publication. All the data will become known and available to the scientific community.
  - Investigators who receive samples and information provided by the Consortium must agree to destroy those samples and information when notified by the T1DGC Coordinating Center or by the NIDDK Central Repository.
  - The Consortium would be recognized in publications in the author line, footnote, or acknowledgement, according to policies developed by the Publications and Presentations Committee. These issues could be sorted out during the review of the application.
4) Samples provided to the Consortium will, at least initially, be deposited in a regional Network repository. Regional Network repositories will initially store samples of DNA, cell lines, plasma, and serum. At timed intervals, and according to a schedule agreed by the Steering Committee and NIDDK, regional Network repositories will ship DNA, cell lines, plasma, and serum samples to the NIDDK Central Repository.

All samples (DNA, plasma, serum, cell lines) will eventually be deposited in the NIDDK Central Repository. These samples will be made available to the scientific community according to policies and a timetable to be determined by the Steering Committee and agreed with NIDDK. Contributing investigators will have an opportunity to provide input as these policies are developed. At the conclusion of the study, access to T1DGC materials will be governed by policies determined by the NIDDK Central Repository.

Study subjects will have the right to request that their samples and information are destroyed at any time in the future. The details of the procedure for this request will be determined by Network policy. The general outline is as follows: the T1DGC Coordinating Center at Wake Forest University will be responsible for notifying the NIDDK Central Repository to destroy requested samples and information. At the end of the study, each regional Network will be responsible for notifying the NIDDK Central Repository to destroy samples and information, when subjects request withdrawal from the study.

Investigators who receive samples and information provided by T1DGC must agree to destroy those samples and information when subjects request withdrawal from the study and upon notification by the T1DGC Coordinating Center or NIDDK Central Repository.

The Consortium will not commercialize the DNA or data deposited with the Consortium. No individual or group will own or claim intellectual property rights to the combined or aggregate Consortium database, other than copyright or similar rights to control use and access to the database or the publication of information and findings generated by the Consortium.

5) The Steering Committee will examine educational and other activities as part of its commitment to establish mechanisms for all interested groups to participate in the activities of the Consortium.
6) The Steering Committee will establish procedures to evaluate opportunities to extend the results of research to develop methods of risk prediction, prevention and therapy in type 1 diabetes. All interested investigators will have an opportunity to provide input as these procedures are developed.

#### Consortium Agreement

I have read the provisions of this Consortium Agreement, and I agree to be bound by its principles.

——————————————————————

Investigator Name

——————————————————————

Investigator Signature

——————————————————————

Date

——————————————————————

Network PI Signature

——————————————————————

Date

## Appendix 2

### Type 1 Diabetes Genetics Consortium Policy Governing Access to Study Repository Samples and Data

This Access Policy applies to T1DGC Contributing Investigators and Consortium Members until the end of the T1DGC study (NIH#U01DK062418). At that time, requests for access to T1DGC samples and data will be governed by policies determined by the NIDDK Central Repository, as specified in the T1DGC Consortium Agreement. All requests from non-members are governed by NIDDK Central Repository policies.

- The study database is frozen on a quarterly basis (January 1, April 1, July 1, and October 1); cumulative data sets can be requested following each freeze, as outlined in this policy and according to the timetable below.
- Samples can be requested by T1DGC investigators twice a year (January–February and July–August).

**Table:**

TIMETABLE:
Samples and data may be requested (for each participant provided) by Contributing Investigators.
**6 months later** →
Access to samples/data may be requested by T1DGC members.
**12 months later** →
Access to samples/data may be requested by non-members.

#### Access Investigator Categories

The T1DGC recognizes two groups of investigators: *T1DGC members*, who have submitted a signed Consortium Agreement to one of the T1DGC Network Centers or the Coordinating Center (including both T1DGC Contributing Investigators and T1DGC investigators who do not contribute samples) and *non-members*, who have not submitted a Consortium Agreement.

- All T1DGC member requests for access to samples or data (excluding requests for participants provided by Contributing Investigators) will be handled by the T1DGC Access Committee.
- All non-member requests for access to samples or data will be handled by application to the NIDDK Central Repository.
- Preference for access to T1DGC holdings is accorded to T1DGC members. T1DGC members may request access to T1DGC samples and data 12 months before non-members may submit similar requests.
- T1DGC members will receive preferential receipt of samples and data.
- The T1DGC website will be used to notify investigators of samples and data available for access applications.

##### Contributing Investigators

Investigators who have contributed samples to T1DGC have the right to request data and samples for each participant provided.

- Requests for quarterly data freezes may be submitted to the investigator’s Regional Network Center at any time.
- Requests for samples may be submitted to the investigator’s Regional Network Center in January–February and July–August.
- Procedures detailed in the ‘Contributing Investigator Request for Samples and Data,’ section of the T1DGC website must be followed.
- Contributing Investigators have priority access to data and samples for each participant they provide. T1DGC will bear the cost for transferring samples to Contributing Investigators.
- Contributing Investigators will have priority receipt of samples and data.

##### Non-contributing Investigators (T1DGC Members)

T1DGC members may request access to data and samples on individuals they did not contribute to the T1DGC six months after these become available to the Contributing Investigator.

##### Investigators Not Members of the T1DGC (Non-Members)

Investigators who are not members of the T1DGC may request access to data and samples (through an application to the NIDDK Central Repository) 12 months after these become available to T1DGC members, or 18 months after these become available to the Contributing Investigator.

#### Renewable and Non-Renewable Resources

There are two types of Consortium resources: renewable and non-renewable. Data and DNA aliquots obtained from cell lines are considered *renewable resources*. Whole-genome amplified DNA, plasma, and serum are considered *non-renewable resources*.

##### Data

Data, referring to information obtained or generated as a result of Consortium activities, are considered a *renewable resource*. Examples of such data include medical information (*e.g.,* age-at-onset), immunological information (*i.e.,* IA2, GAD65 autoantibodies), and genetic information (*e.g.,* genome scan linkage data, HLA genotype data, *INS*, *CTLA4*). T1DGC databases include results from T1DGC-directed genetic analyses, laboratory assays of serum and plasma specimens, and data collected on T1DGC forms.

##### Samples

Samples, referring to biological materials obtained from blood, include cell lines, genomic DNA, whole genome amplified DNA, plasma, and serum.

- Only Contributing Investigators are entitled to receive a cell line aliquot from samples that they submit, and only for use that is consistent with any restrictions arising from informed consent. Cell lines will not otherwise be distributed by the T1DGC.
- There are different criteria for access to renewable versus non-renewable samples. There are also different review and approval procedures (see below).
- Because access depends on sample availability, requests for access to samples may be made only according to a determined schedule. This applies to both renewable and non-renewable sample resources.
- Access to each sample will be governed by the provisions of its associated informed consent form. This means that some samples may be restricted to non-commercial use. Ordinarily, investigators who receive access to samples will also receive access to the data associated with those samples. However, data may become available to the investigator on a different time schedule than the samples.
- The timing of release of non-renewable sample resources is likely to be different from the timing of release of renewable samples.
- The investigator who receives access to T1DGC samples will bear the costs for sample transfer.

#### Applying for Access to Renewable and Non-Renewable Resources

To apply for access to T1DGC samples and/or data, complete the Access Application form posted in the ‘Application for Access to T1DGC Data and Sample,’ section of the T1DGC website. NOTE: The Access Application form is Appendix 2.A of this document (.pdf version) and is also posted on the T1DGC website (.doc version).

Applications must be submitted to the Access Committee via the web-based Access System. A Confidentiality Certification (Appendix 2.B) for all individuals with access to the data or samples should be completed and submitted to the Regional Network Center or Coordinating Center (for T1DGC members).

The timetable for applying for access to specified T1DGC resources depends on T1DGC membership (see TIMETABLE above and/or the T1DGC website).

##### General Guidelines

- All applications will be reviewed by the Access Committee for concordance with the aims of the T1DGC and security.
- The Coordinating Center will keep confidential records of all applications and will provide the Steering Committee regular summary reports of its activities. For non-renewable applications, the evaluation forms from each Access Member and the written critique from the Access Committee Chair will be the sole record of the deliberations.
- Access is conditional on availability of samples and/or data, and agreement to abide by T1DGC policies related to publication, specimen disposal, custodianship, ethical approval and informed consent, patenting, and confidentiality.
- Access to each sample will be governed by the provisions of its associated informed consent form. Access to samples from particular countries is subject to the laws pertaining to those countries; individuals requesting access to samples will be required to sign an agreement acknowledging that they understand and will comply with any country-specific restrictions associated with certain samples. A plan for compliance will be required for the application requesting access to restricted samples.
- Access to T1DGC samples and/or data is conditional on the investigator agreeing to submit results to the NIDDK Central Repository for incorporation among the T1DGC data holdings, which enhances the scientific value of these samples and extends opportunities for collaborative research. This reporting must occur within one year after receipt of the samples and/or data.
- The Access Request system will simultaneously notify the Coordinating Center of all successful applications so that it can begin the process of data and/or sample transfer. Transfer will be accompanied by documentation related to sample collection and storage. Transfer of required data from the T1DGC database will be negotiated directly with the applicants. The Coordinating Center will regularly report to the Access Committee on the status of sample and data transfers.
- Unsuccessful applicants may revise their application and re-apply to the Access Committee. If the application is denied a second time, the unsuccessful applicant may appeal the decision. An appeal will be considered only if the review process was flawed. The Chair of the Steering Committee will determine if the review was flawed and, if so, the Steering Committee will constitute a separate review group to handle the appeal and review the application.
- The T1DGC Publications and Presentations Committee will track publications derived from T1DGC samples and data. Investigators granted access to samples and data will be requested to provide updated information on such publications to that Committee. Study publications policy (set by the T1DGC Publications and Presentations Committee) will govern the citation and release of this information to the general public.
- The T1DGC Coordinating Center maintains summary reports of its databases on the password-protected study website. These include, in aggregate, distributions of genetic and phenotypic characteristics of the study cohort and data related to the collection and processing of these samples. The nature and content of these reports are determined by T1DGC committees, its sponsors, and its External Evaluation Committee.

#### Approval Process for Renewable Resources

For *renewable* sample resources, the main criterion for approval of applications is scientific appropriateness.

- Applications for renewable samples will be considered approved if approved by majority vote of six members of the Access Committee. Applications will be decided within four weeks of receipt. The Access Committee Chair will notify applicants of the final decision.
- Approval for access to subsequent versions of the T1DGC database may be requested through an extension of the original access request. Extension of a data request is permissible only if the specific aims of the original request have not changed since the time of the initial submission. If the specific aims have changed, a new access request must be submitted.

#### Approval Process for Non-Renewable Resources

For *non-renewable* sample resources, the main criterion for approval of applications is scientific merit; additional criteria considered by the Access Committee are outlined below.

1. The Access Committee (which includes a representative from the Coordinating Center and the funding agencies) will review each application for access to non-renewable samples, using the form developed for this purpose (Appendix 2.C). In addition, one outside reviewer will be appointed by the Chair of the Access Committee. A primary reviewer will be appointed in each review panel, using the NIH model. The T1DGC Coordinating Center representative will be responsible for evaluating the logistical needs of the application and confirming the availability of the requested samples.
2. Factors considered in the review of applications for non-renewable specimens include the scientific merit of the application, its uniqueness and potential contribution, synergy with the goals of the T1DGC, and the research experience of the applicant. Overlapping applications may result in recommendation that collaborations be implemented.
3. Applications for non-renewable resources should:
  - clearly state the hypothesis to be tested and the number of samples needed to achieve this (*i.e.,* power calculations);
  - state precisely what assays will be undertaken, by what methods and the amount of sample required for each;
  - contain an undertaking not to use the sample for any other purpose without prior authorization;
  - include an undertaking to return all unused sample by a given deadline; and
  - explain why T1DGC samples are needed (*i.e.,* why no other resources can address the hypothesis).
4. Applications for non-renewable samples will be considered approved if approved by a two-thirds majority of the Access Committee (including the external reviewer). Applications will be decided within 12 weeks of receipt. The review panel will develop a written critique of each application and a rationale for its decision. The Access Committee Chair will notify applicants of the final decision.

## Appendix 2.A

### T1DGC Application for Access to Data and Samples

**Table:**

**Date of submission:**
**Resource Requested: (Mark all that apply.)**
**Renewable Resources:**
□ **DNA (5 mcg aliquot)**
□ **Data (Specify requested data) set[s**]: _______________________________________
**NOTE: Data set name is a required field. See list of “Available Data Sets and Samples” under “Access to T1DGC Data and Samples” link on****www.t1dgc.org**.
**Non-Renewable Resources:**
□ **Whole Genome Amplified DNA (5 mcg aliquot)**
□ **Serum (0.5 mL aliquot)**
□ **Plasma (0.5 mL aliquot)**
**Project title:**
**Corresponding investigator and full contact information:** Name: Address: Telephone: FAX: E-mail:
**Name(s), affiliation(s) and address(es) of major co-investigator(s) and/or collaborator(s):**
**Name(s), affiliation(s) and address(es) of project analyst(s):**
**Abstract (250 words or less):**
**Specific aims:**
**Previous peer review of the project.** Indicate whether the genetic components of the project have undergone previous peer review, and by whom. Indicate the outcome of the review. If an application is currently pending at the NIH, provide details about the study section and institute assignment, if known.
**Source(s) of funds for the project.** If no new funds are required, this should be stated. If funded by the NIH, list the sponsoring institute and the dates of support. If approval is sought conditional on the applicant’s success in obtaining funding, a specific timeline for this must be provided.
**Number of samples to be analyzed and the projected timeline for obtaining the samples from T1DGC.** (For non-renewable resources, a formal justification for the requested number of samples must be provided, including power analyses. This is critical for access to the limited plasma and serum samples.)
**Brief outline of the plan for the next phase of the project if linkage or association is found (if applicable).** Include specific plans for isolating the locus (loci) and name the individuals responsible for each step. Attach letters of collaboration from these individuals.
**Description of core data required from the T1DGC central databases, including process and phenotypic data.**
**Measures to ensure the security of specimens and data.** In addition, plans for disposal of any unused specimens and data must be described.
**Background information about the disease/trait including the rationale for carrying out this particular study.** Describe any unique features about the disease/trait that would single out this project for special consideration.
**Analysis strategy for the resulting information and choice of analytic methods and software.** If collaborations are established for analytical services, include letters of collaboration.
**Assurance that the project has been reviewed for human subject protection by an appropriate Institutional Review Board (IRB) or Ethics Committee (EC).**
**Description of any commercial aims and likely benefits ensuing from the project.** Details of pending or granted patents relevant to the application must be provided.
**If samples are requested, please provide shipping contact information.**
Name of Contact:
Shipping Address:
E-mail Address:
Phone Number:
**Have all of co-investigators and collaborators approved the final version of this application?** □ YES □ NO
**Is there a deadline for submission of this material to an external agency?** □ YES □ NO
**If yes, what is the deadline and when would you like comments back? __________/__________/___________**
**DD/MM/YYYY**
**I have read and agree to abide by the T1DGC Access Policy, the T1DGC Publications and Presentations Policy, and the consent guidelines conferred by study participants.**
□ YES □ NO
**I have signed and submitted the T1DGC Confidentiality Certification to the Network Center or the Coordinating Center.**
□ YES □ NO
**All individuals who will have access to the data and/or samples have submitted the T1DGC Confidentiality Certification to the Network Center or the Coordinating Center.**
□ YES □ NO
**I agree to submit all results from analysis of these samples to the NIDDK Central Repository for incorporation among the T1DGC data holdings (including information on quality control methods).**
□ YES □ NO
**I understand that some samples will be restricted to non-commercial use only.**
□ YES □ NO
**Applications for non-renewable resources must include submission of the following items:**
**• CV(s) for key personnel involved in the project (NIH format required)**
**• Letter(s) of support/commitment from major collaborator(s) and/or co-investigator(s)**
**• Essential reprints or preprints (no more than 3)**
**Submission of these items is optional for applications for renewable resources.**

## Appendix 2.B

### T1DGC Confidentiality Certification

All individuals with any access to Type 1 Diabetes Genetics Consortium (T1DGC) data must sign a Confidentiality Certification. This includes, but is not limited to, the following groups of individuals:

- Principal Investigators
- Co-Investigators and Investigators
- Coordinating Center staff, including any data entry, data management, data analysis and support staff
- Regional Network staff and support staff
- Field Center staff and support staff
- Laboratory staff and support staff
- Data analysts (on-site and off-site)
- Fellows and students
- Consultants
- Ancillary study investigators

Each individual with access to T1DGC data must read, sign, and date the Confidentiality Certification. The Network Principal Investigator must also sign the certification and keep the original copy on file at the Regional Network Center. A copy of the completed certification should be forwarded to the Coordinating Center.

The Principal Investigators (Field Centers, Regional Networks, and Coordinating Center) are responsible for ensuring that all individuals currently affiliated with the T1DGC Study through their site sign the Confidentiality Certification. The Coordinating Center is specifically responsible for ensuring that all subcontractors and consultants to the Study through the Coordinating Center contract, including laboratory investigators and staff, sign the Confidentiality Certification. The Principal Investigators are responsible for ensuring that all individuals who become affiliated with the T1DGC Study through employment or consulting in the future sign the Confidentiality Certification at the time he/she joins the Study.

### Type 1 Diabetes Genetics Consortium Confidentiality Certification

As an employee of, consultant to, or fellow/student involved with the Type 1 Diabetes Genetics Consortium (T1DGC) Study funded by the National Institute of Diabetes and Digestive and Kidney Diseases (NIDDK), I am aware of the confidential nature of data on research participants maintained by the Study, and the necessity of maintaining that confidentiality.

I agree not to **transfer** or **disclose** any confidential data, nor any information about individual T1DGC Study participants, except as necessary for data/safety monitoring or programmatic management, in the course of my responsibilities at work nor in private, either during or after my affiliation with the T1DGC Study. I agree not to transfer any T1DGC data or biological specimens to individuals outside the T1DGC Study Group without the written permission of the T1DGC Steering Committee. Further, I agree to return all T1DGC data to the Principal Investigator or delete/destroy all T1DGC data upon termination of my affiliation with the Study.

I understand that as an employee of, consultant to, or fellow/student involved with the T1DGC study, I am subject to the provisions of laws and regulations related to confidentiality of study data in the country where the work is performed.

Name (print): __________________________________

Signature: ______________________________________

Date: __________________________________________

T1DGC Site: ____________________________________

Principal Investigator’s Signature: ________________

Date: __________________________________________

## Appendix 2.C

**T1DGC Evaluation Form: Non-Renewable Resource Request**

**Access Request Number: AR**

**Please rate items 1–5 as “Acceptable” or “Not Acceptable”. All comments will be forwarded to the investigator submitting the request.**

1) Importance of the scientific question to type 1 diabetes research:
  Acceptable _________ Not Acceptable _________
  Comments:
2) Unique requirement for samples requested:
  Acceptable __________ Not Acceptable _________
  Comments:
3) Quality and thoroughness of the proposal in outlining the specific hypotheses and methods:
  Acceptable _________ Not Acceptable _________
  Comments:
4) Number of samples and amount of sample required:
  Acceptable __________ Not Acceptable _________
  Comments:
5) Plan for sharing data results with other investigators:
  Acceptable _________ Not Acceptable _________
  Comments:
6) Additional comments or questions:

———————————————————————–

**Request Approved: __________ Request Not Approved: ___________**

## Appendix 3

### Type 1 Diabetes Genetics Consortium

**Asia-Pacific Network:** Tracey Baskerville (Mater Children’s Hospital, Australia); Nines Bautista (Institute for Study on Diabetes Foundation, Philippines); Eesh Bhatia (Sanjay Gandhi Postgraduate Institute, India); Vijayalakshmi Bhatia (Sanjay Gandhi Postgraduate Institute, India); Kamaruzaman Bin Hasan (National University of Malaysia Hospital, Malaysia); Francois Bonnici (University of Cape Town, South Africa); Thomas Brodnicki (Walter & Eliza Hall Institute of Medical Research, Australia); Brian Browning (The University of Auckland, New Zealand); Fergus Cameron (Royal Children’s Hospital, Australia); Katharee Chaichanwatanakul (Mahidol University, Thailand); Pik To Cheung (Queen Mary Hospital, Hong Kong); Peter Colman^2,5,11,15^ (Walter & Eliza Hall Institute of Medical Research, Australia); Andrew Cotterill (Mater Children’s Hospital, Australia); Jenny Couper (Women’s and Children’s Hospital, Australia); Patricia Crock (John Hunter Children’s Hospital, Australia); Ric Cutfield (North Shore Hospital, New Zealand); Tim Davis (Fremantle Hospital, Australia); Paul Dixon (Diabetes Lifestyle Centre, New Zealand); Kim Donaghue (Children’s Hospital at Westmead, Australia); Katrina Dowling^4^ (Australian Red Cross Blood Service, Australia); Paul Drury (Auckland Diabetes Centre, New Zealand); Sarah Dye (Western Australia Institute for Medical Research, Australia); Shane Gellert^2^ (The Royal Melbourne Hospital, Australia); Rohana Abdul Ghani (National University of Malaysia Hospital, Malaysia); Ristan Greer (University of Queensland, Australia); Xueyao Han (Peking University People’s Hospital, China); Len Harrison (Walter & Eliza Hall Institute of Medical Research, Australia); Nick Homatopoulos^4^ (Australian Red Cross Blood Service, Australia); Linong Ji (Peking University People’s Hospital, China); Tim Jones (Princess Margaret Hospital for Children, Australia); Loke Kah Yin (Children’s Medical Institute, Singapore); Nor Azmi Kamaruddin (National University of Malaysia Hospital, Malaysia); Uma Kanga (All India Institute of Medical Sciences, India); Alok Kanungo (Cuttack Diabetes Research Foundation, India); Gurvinder Kaur (All India Institute of Medical Sciences, India); Betty Kek (Children’s Medical Institute, Singapore); Simon Knowles^4^ (Australian Red Cross Blood Service, Australia); Jeremy Krebs (The Diabetes Centre, New Zealand); Neeraj Kumar (All India Institute of Medical Sciences, India); Yann-Jinn Lee^7^ (Mackay Memorial Hospital, Taiwan); Xiaoying Li (Shanghai Jiao-Tong University, China); Supawadee Liktimaskul (Mahidol University, Thailand); Margaret Lloyd (Children’s Hospital at Westmead, Australia); Amanda Loth^5,10^ (Walter & Eliza Hall Institute of Medical Research, Australia); Anthony Louey^3,4^ (Australian Red Cross Blood Service, Australia); Narinder Mehra^8^ (All India Institute of Medical Sciences, India); Tony Merriman (University of Otago, New Zealand); Liu Min (Beijing Children’s Hospital, China); Grant Morahan^1,9,12,14,15^ (Western Australia Institute for Medical Research, Australia); Robert Moses (Illawarra Diabetes Services, Australia); Grant Mraz^4^ (Australian Red Cross Blood Service, Australia); Rinki Murphy (Auckland Diabetes Centre, New Zealand); Ian Nicholson^4^ (Australian Red Cross Blood Service, Australia); Araceli Panelo (Institute for Studies on Diabetes Foundation, Philippines); Perlita Poh^2^ (Royal Melbourne Hospital, Australia); Gareth Price (Mater Medical Research Institute, Australia); Nirubasini Ratnam (Princess Margaret Hospital for Children, Australia); Carani Sanjeevi^6^ (Karolinska Hospital, Sweden); Saikiran Sedimbi (Karolinska Hospital, Sweden); Shuixian Shen (Fudan University, China); Goh Siok Ying (The Children’s Medical Institute, Singapore); Brian Tait^3,4,5,6^ (Australian Red Cross Blood Service, Australia); Nikhil Tandon (All India Institute of Medical Sciences, India); Allison Thomas (Walter & Eliza Hall Institute of Medical Research, Australia); Mike Varney^3,4^ (Australian Red Cross Blood Service, Australia); Praewvarin Weerakulwattana (Mahidol University, Thailand); Jinny Willis (Christchurch Hospital, New Zealand.

**European Network:** Elvis Abang Akwo (Yaounde Central Hospital, Cameroon); Lotte Albret^5,10^ (Hagedorn Research Institute and Steno Diabetes Center, Denmark); Francisco Ampudia-Blasco (Clinic University Hospital Valencia, Spain); Jesus Argente (Hospital Infantil Universitario Nino Jesus, Spain); Magdalena Avbelj (University Children’s Hospital, Slovenia); Gulja Babadjanova (Moscow State Medical University, Russia); Klaus Badenhoop^14^ (University Clinic Frankfurt/Main, Germany); Tadej Battelino (University Children’s Hospital, Slovenia); Georg Beilhack^14^ (University of Ulm, Germany); Regine Bergholdt (Hagedorn Research Institute and Steno Diabetes Center, Denmark); Polly Bingley^2,5^ (University of Bristol, United Kingdom); Bernhard Boehm^4,5,14^ (Ulm University, Germany); Jo Bolidson^2^ (University of Bristol, United Kingdom); Kerstin Brismar (Karolinska Hospital, Sweden); Caroline Brorsson^14^ (Hagedorn Research Institute and Steno Diabetes Center, Denmark); Joyce Carlson^3,5^ (University Hospital MAS, Sweden); Luis Castano (Hospital de Cruces, Spain); Kyla Chandler^2^ (University of Bristol, United Kingdom); Valentino Cherubini (Salesi Hospital, Italy); Ondrej Cinek (Motol University Hospital, Czech Republic); Elisa Cipponeri (University Campus Bio-Medico, Italy); Raquel Corripio Collado (Consorci Sanitari Parc Tauli, Spain); Alberto de Leiva (Hospital Sant Pau, Spain); Iveta Dzivite (University Children’s Hospital, Latvia); Ana Fagulha (University Hospital, Portugal); Merce Fernandez Balcells (Hospital Trueta, Spain); Beatriz Garcia Cuartero (Hospital Severo Ochoa, Spain); Concepcion Garcia Lacalle (Hospital Severo Ochoa, Spain); Cristian Guja (Institute of Diabetes, Nutrition & Metabolic Diseases, Romania); Pilar Gutiérrez (Hospital Universitario de Getafe, Spain); Alona Hamou (Schneider Children’s Medical Center of Israel, Israel); Erifili Hatziagelaki (University of Athens, Greece); Simon Heath^7^ (Centre National de Genotypage, France); Kaire Heilman (Tartu University Children’s Hospital, Estonia); Wolfgang Helmberg^5,7^ (Medical University Graz, Austria); Orna Hermon (Schneider Children’s Medical Center of Israel, Israel); Marta Hernandez (Hospital Universitari Arnau de Vilanova and Hospital Universitario de Canarias, Spain); Iris Holzheu^4^ (Ulm University, Germany); Nora Hosszufalusi (Semmelweis University, Hungary); Jorma Ilonen (University of Turku, Finland); Constantin Ionescu-Tirgoviste (Institute of Diabetes, Romania); Jesper Johannesen (Steno Diabetes Center, Denmark); Cecile Julier^1,9,12,14^ (Centre National de Genotypage, France); Heinrich Kahles^14^ (Klinikum der J.W. Goethe-Universität, Germany); Ida Kinalska (Medical University of Bialystok, Poland); Mikael Knip (University of Helsinki, Finland); Ingrid Kockum^7,14^ (Karolinska Hospital, Sweden); Eija Kojo (University of Helsinki, Finland); Olga Kordonouri (Children’s Hospital auf der Bult, Germany); Adam Kretowski (Medical University of Bialystok, Poland); Dora Krikovszky (Semmelweis University, Hungary); Angelika Kurkhaus^4^ (Ulm University, Germany); Madiusz Kuzmicki (Medical University of Bialystok, Poland); Eva Lavant^3^ (University Hospital MAS, Sweden); Anna Long^2^ (University of Bristol, United Kingdom); Johnny Ludvigsson (University Hospital, Sweden); Laszlo Madacsy (Semmelweis University, Hungary); Katarzyna Maliszewska (Medical University of Bialystok, Poland); Mara Marga (P. Stradins University Hospital, Latvia); Marissa Penna Martinez (University Clinic Frankfurt/Main, Germany); Didac Mauricio^6^ (Hospital Universitari Arnau de Vilanova and Hospital Sant Pau, Spain); Gertrud Mazurkievicz^4^ (Ulm University, Germany); Jorn Nerup^1,15^ (Steno Diabetes Center, Denmark); Antanas Norkus (Institute of Endocrinology of Kaunas University of Medicine, Lithuania); Francisco Javier Novoa Mogollon (Hospital Universitario Insular, Spain); Anna Okruszko (Medical University of Bialystok, Poland); Chiara Pettinari (Salesi Hospital, Italy); Moshe Phillip (Schneider Children’s Medical Center of Israel, Israel); Valdis Pirags (P. Stradins University Hospital, Latvia); Flemming Pociot^1,11,12,14,15^ (Hagedorn Research Institute and Steno Diabetes Center, Denmark); Paolo Pozzilli (University Campus Bio-Medico, Italy); Radu Racasan (University Clinic Frankfurt/Main, Germany); Klemens Raile (Vircow Clinic Charité Berlin, Germany); Rebecca Rappner^3^ (University Hospital MAS, Sweden); Maria Jesus Rodriguez Troyano (University Hospital of Las Palmas de Gran Canaria, Spain); Bart O. Roep (Leiden University Medical Center, Netherlands); Saba Rokni^2^ (Southmead Hospital, United Kingdom); Silke Rosinger^4^ (Ulm University, Germany); Oscar Rubio-Cabezas (Hospital Infantil Universitario Nino Jesus, Spain); Christa Ruckgaber^4^ (Ulm University, Germany); Ilhan Satman (Istanbul University, Turkey); Edith Schober (University Children’s Hospital, Austria); Jochen Seufert (Medizinsche Poliklinik der Universität, Germany); Rosi Sing^4^ (Ulm University, Germany); Jan Skrha (Faculty of Medicine 1, Czech Republic); Eugene Sobngwi (Central National Obesity Centre and Hospital of Diabetes Endocrine, Cameroon); Michelle Somerville^2^ (University of Bristol, United Kingdom); Giatgen Spinas^8^ (University Hospital, Switzerland); Zdenek Sumnik (University Hospital Motol, Czech Republic); Vallo Tilmann (Tartu University Children’s Hospital, Estonia); Dag Undlien^6^ (University of Oslo, Norway); Vaidotas Urbanavicius (Vilnius University Hospital, Lithuania); Bart Van der Auwera^8^ (Vrije Universiteit Brussel, Belgium); Federico Vasquez San Miguel (Hospital de Cruces, Spain); Andriani Vazeo-Gerasimidi (Diabetes Center P&A Kyriakou Children’s Hospital, Greece); Dzilda Velickiene (Institute of Endocrinology of Kaunas University of Medicine, Lithuania); Ana Wägner^5,10^ (University Hospital of Las Palmas de Gran Canaria, Spain, and Steno Diabetes Center, Denmark); Markus Walter (Diabetes Research Institute, Germany); Alistair Williams^2^ (University of Bristol, United Kingdom); Anette Ziegler (Diabetes Research Institute, Germany).

**North American Network:** Matthew Agleham^3^ (Roche Molecular Systems, United States); Alan Aldrich^5,10^ (University of Alaska Anchorage College of Arts & Sciences, United States); Ramin Alemzadeh (Medical College of Wisconsin, United States); Chester Alper (Immune Disease Institute, United States); Theresa Aly (Barbara Davis Center for Childhood Diabetes, United States); Dimitris Anastassiou (Columbia University, United States); Shaily Arora^3^ (Children’s Hospital Oakland Research Institute, United States); Audrey Austin (Children’s National Medical Center, United States); Dorothy Becker (Rangos Research Center, United States); Christophe Benoist (Joslin Diabetes Center, United States); Noureddine Berka^6^ (Calgary Laboratory Services, Canada); Suruchi Bhatia (Oakland Children’s Hospital Research Center, United States); Persia Bonella^3^ (Roche Molecular Systems, United States); Nunzio Bottini^14^ (University of Southern California, United States); Sean Boyle^3^ (Roche Molecular Systems, United States); Jeanah Braden (Children’s Hospital Oakland Research Institute, United States); Barry Brady (Arkansas Children’s Hospital, United States); Wendy Brickman (Children’s Memorial Hospital, United States); Richard Christensen (Humphreys Diabetes Center, United States); Patrick Concannon^1,9,12,14^ (University of Virginia, United States); Robert Couch (University of Alberta, Canada); Debra Counts (University of Maryland, United States); Jill Crandall (Albert Einstein College of Medicine, United States); Mark Daniels (Children’s Hospital of Orange County, United States; Larry Dolan (Cincinnati Children’s Hospital Medical Center, United States); David Donaldson (Utah Diabetes Center, United States); Alessandro Doria^6^ (Joslin Diabetes Center, United States); George Eisenbarth^2,5,13,14^ (Barbara Davis Center for Childhood Diabetes, United States); James Elder (University of Michigan, United States); Rita El-Hajj (Main Line Health Heart Center, United States); Henry Erlich^1,3,5,13,14^ (Roche Molecular Systems, United States); Pamela Fain (Barbara Davis Center for Childhood Diabetes, United States); Anna Lisa Fear^3^ (Children’s Hospital Oakland Research Institute, United States); Robert Ferry (The University of Texas Health Science Center at San Antonio, United States); Rosanna Fiallo-Scharer (Barbara Davis Center for Childhood Diabetes, United States); Daniel Geraghty (Fred Hutchinson Cancer Research Center, United States); Soumitra Ghosh^6^ (Medical College of Wisconsin, United States); Steven Gitelman (University of California at San Francisco, United States); Michelle Godwin^4^ (Fred Hutchinson Cancer Research Center, United States); Robin Goland (Naomi Berrie Diabetes Center, United States); Nathan Goodman^7^ (Institute for Systems Biology, United States); Greg Goodwin (Joslin Diabetes Center, United States); Jenna Gravely^4^ (Fred Hutchinson Cancer Research Center, United States); Carla Greenbaum^8,11,15^ (Benaroya Research Institute, United States); Chelsea Gudgeon^4^ (Fred Hutchinson Cancer Research Center, United States); Fred Gunville (Billings Clinic, United States); William Hagopian^11^ (University of Washington, United States); Hakon Hakonarson (Children’s Hospital of Philadelphia, United States); John Hansen^4,5^ (Fred Hutchinson Cancer Research Center, United States); Kimberly Harrington^4^ (Fred Hutchinson Cancer Research Center, United States); Jeanne Hassing (Sacred Heart, United States); Wendy Hilliker^4^ (Fred Hutchinson Cancer Research Center, United States); Robert Hoffman (Ohio State University, United States); Erin Hulbert (Institute for Systems Biology, United States); Roberto Izquierdo (SUNY Upstate Medical University, United States); Nicholas Jospe (University of Rochester, United States); Kevin Kaiserman (Children’s Hospital Los Angeles, United States); Francine Kaufman (Children’s Hospital Los Angeles, United States); Samuel Kim^3^ (Roche Molecular Systems, United States); Erin Kloos^4^ (Fred Hutchinson Cancer Research Center, United States); Roman Kosoy (Benaroya Research Institute, United States); James Lane (University of Nebraska, United States); Julie Lane^3^ (Children’s Hospital Oakland Research Institute, United States); Jean Lawrence (Kaiser Permanente, United States); Claresa Levetan (Main Line Health Heart Center, United States); Phil Levin (MODEL Clinical Research, United States); Rebecca Lipton (University of Chicago, United States); John Lonsdale (Human Biological Data Interchange, United States); Victoria Magnuson (Children’s Hospital of Wisconsin, United States); Jennifer Marks (University of Miami, United States); Beth Mayer-Davis (University of South Carolina, United States); Robert McEvoy (Children’s Hospital of Minnesota, United States); Richard McIndoe^7^ (Medical College of Georgia, United States); Lesley Merkle^4^ (Fred Hutchinson Cancer Research Center, United States); Daniel Metzger (BC Children’s Hospital, Canada); Dongmei Miao^2^ (Barbara Davis Center for Childhood Diabetes, United States); Eric Mickelson^4^ (Fred Hutchinson Cancer Research Center, United States); Priscilla Moonsamy^3^ (Roche Molecular Systems, United States); Wayne Moore (Children’s Mercy Hospital, United States); Antoinette Moran (University of Minnesota, United States); Janelle Noble^3,5,13,14^ (Children’s Hospital Oakland Research Institute, United States); Gary Olsem^4^ (Fred Hutchinson Cancer Research Center, United States); Suna Onengut-Gumuscu^14^ (University of Virginia, United States); Tihamer Orban (Joslin Diabetes Center, United States); Craig Orlowski (University of Rochester Medical Center, United States); Andrew Paterson (University of Toronto, Canada); Massimo Pietropaolo (University of Michigan Medical School, United States); Catherine Pihoker (Children’s Hospital and Regional Medical Center, United States); Constantin Polychronakos^11,14^ (McGill University Health Center, Canada); Jeff Post^3^ (Roche Molecular Systems, United States); Daniel Postellon (Helen DeVos Children’s Hospital, United States); Alberto Pugliese^9,14^ (University of Miami, United States); HuiQi Qu^14^ (Montreal Children’s Hospital, Canada); Teresa Quattrin (Women and Children’s Hospital of Buffalo, United States); Mark Rappaport (Pediatric Endocrine Associates, United States); Philip Raskin (University of Texas Southwestern Medical Center, United States); Heather Risbeck^4^ (Fred Hutchinson Cancer Research Center, United States); Henry Rodriguez (Riley Hospital for Children, United States); Luisa Rodriguez (Baylor College of Medicine, United States); Michelle Rogers^4^ (Fred Hutchinson Cancer Research Center, United States); Leticia Rubalcava (Children’s Hospital Oakland Research Institute, United States); Bill Russell (Vanderbilt University, United States); Desmond Schatz (University of Florida, United States); Carla Scott (University of Texas Health Science Center at San Antonio, United States); Jin-Xiong She^14^ (Medical College of Georgia, United States); Heather Shilling (Benaroya Research Institute, United States); Dorothy Shulman (University of South Florida, United States); Leslie Soyka (University of Massachusetts Memorial Center, United States); Phyllis Speiser (Schneider Children’s Hospital, United States); Harold Starkman (Atlantic Health System, United States); Andrea Steck^14^ (Barbara Davis Center for Childhood Diabetes, United States); Sarah Stender (University of Tennessee, United States); Lorraine Stratton (University of Arizona, United States); Daniel Sur^3^ (Roche Molecular Systems, United States); Shayne Taback (University of Manitoba, United States); Kathryn Thrailkill (Arkansas Children’s Hospital, United States); Ellen Toth (University of Alberta, Canada); Patricia Trymbiski (Doylestown Hospital, United States); Eva Tsalikian (University of Iowa, United States); Katherine Vertachnik^4^ (Fred Hutchinson Cancer Research Center, United States); Jack Wahlen (Endocrine Research Specialists, United States); Xujing Wang (Max McGee National Research Center of Juvenile Diabetes, United States); Sandra Weber (Greenville Hospital System, United States); Diane Wherrett (Hospital for Sick Children, Canada); Steven Willi (Children’s Hospital of Phildelphia, United States); Darrell Wilson (Stanford University, United States); Jerry Youkey (Greenville Hospital System, United States); Neal Young (National Institutes of Health, United States); Liping Yu^2^ (Barbara Davis Center for Childhood Diabetes, United States); Lue Ping Zhao (Fred Hutchinson Cancer Research Institute, United States); Donald Zimmerman (Children’s Memorial Hospital, United States).

**United Kingdom Network:** Ellen Adlem^4^ (University of Cambridge, United Kingdom); James Allen^4^ (University of Cambridge, United Kingdom); Jeffrey Barrett (University of Cambridge, United Kingdom); Judy Brown^4^ (University of Cambridge, United Kingdom); Oliver Burren^4^ (University of Cambridge, United Kingdom); Pamela Clarke^4^ (University of Cambridge, United Kingdom); David Clayton^4^ (University of Cambridge, United Kingdom); Gillian Coleman^4^ (University of Cambridge, United Kingdom); Jason Cooper^4^ (University of Cambridge, United Kingdom); Francesco Cucca^6^ (University of Sassari, United Kingdom); Lucy Davison (University of Cambridge, United Kingdom); Kate Downes (University of Cambridge, United Kingdom); Simon Duley^4^ (University of Cambridge, United Kingdom); David Dunger^11^ (University of Cambridge, United Kingdom); Laura Esposito (University of Cambridge, United Kingdom); Vin Everett^4^ (University of Cambridge, United Kingdom); Sarah Field (University of Cambridge, United Kingdom); Jason Hafler (University of Cambridge, United Kingdom); Matthew Hardy^4^ (University of Cambridge, United Kingdom); Deborah Harrison^4^ (University of Cambridge, United Kingdom); Inge Harrison^4^ (University of Cambridge, United Kingdom); Steve Hawkins^4^ (University of Cambridge, United Kingdom); Barry Healy^4^ (University of Cambridge, United Kingdom); Simon Hood^4^ (University of Cambridge, United Kingdom); Simon Howell^8^ (King’s College, United Kingdom); Joanna Howson (University of Cambridge, United Kingdom); Meeta Maisuria^4^ (University of Cambridge, United Kingdom); William Meadows^4^ (University of Cambridge, United Kingdom); Trupti Mistry^4^ (University of Cambridge, United Kingdom); Sergey Nezhenstsev (University of Cambridge, United Kingdom); Sarah Nutland^4,5^ (University of Cambridge, United Kingdom); Nigel Ovington^4^ (University of Cambridge, United Kingdom); Vincent Plagnol (University of Cambridge, United Kingdom); Dan Rainbow (University of Cambridge, United Kingdom); Kara Rainbow (University of Cambridge, United Kingdom); Srilakshmi Raj (University of Cambridge, United Kingdom); Helen Schuilenburg^4^ (University of Cambridge, United Kingdom); Anna Simpson^4^ (University of Cambridge, United Kingdom); Luc Smink^7^ (University of Cambridge, United Kingdom); Debbie Smyth (University of Cambridge, United Kingdom); Helen Stevens^4^ (University of Cambridge, United Kingdom); Niall Taylor^4^ (University of Cambridge, United Kingdom); John Todd^1,4,12,14,15^ (University of Cambridge, United Kingdom); Jaakko Tuomilehto (National Public Health Institute, Finland); Neil Walker^4,5^ (University of Cambridge, United Kingdom); Linda Wicker (University of Cambridge, United Kingdom); Barry Widmer^4^ (University of Cambridge, United Kingdom); Mark Wilson^4^ (University of Cambridge, United Kingdom); Heather Withers^5,10^ (University of Cambridge, United Kingdom); Jennie Yang (University of Cambridge, United Kingdom).

**Coordinating Center:** Mark Brown (Wake Forest University Health Sciences, United States); Wei-Min Chen (University of Virginia, United States); Arnetta Crews (Wake Forest University Health Sciences, United States); Jason Griffin (Wake Forest University Health Sciences, United States); Mark Hall^8^ (Wake Forest University Health Sciences, United States); Teresa Harnish (Wake Forest University Health Sciences, United States); John Hepler (Wake Forest University Health Sciences, United States); Joan Hilner^5,8,10^ (University of Alabama at Birmingham, United States); Nancy King^8^ (Wake Forest University Health Sciences, United States); Kurt Lohman (Wake Forest University Health Sciences, United States); Lingyi Lu (Wake Forest University Health Sciences, United States); Josyf Mychaleckyj^5,7^ (University of Virginia, United States); Jay Nail (Wake Forest University Health Sciences, United States); Letitia Perdue^5,10^ (Wake Forest University Health Sciences, United States); June Pierce (Wake Forest University Health Sciences, United States); David Reboussin^5,6^ (Wake Forest University Health Sciences, United States); Stephen Rich^1,12,14^ (University of Virginia, United States); Scott Rushing (Wake Forest University Health Sciences, United States); Michele Sale (University of Virginia, United States); Elizabeth Sides^5,10^ (Wake Forest University Health Sciences, United States); Beverly Snively^11^ (Wake Forest University Health Sciences, United States); Hoa Teuschler (Wake Forest University Health Sciences, United States); Goodrich Theil (Wake Forest University Health Sciences, United States); Lynne Wagenknecht (Wake Forest University Health Sciences, United States); Dustin Williams (Wake Forest University Health Sciences, United States).

**Project Office:** Beena Akolkar^1,5,6,9,12^ (National Institute of Diabetes and Digestive and Kidney Diseases/National Institutes of Health, United States); Catherine McKeon^8^ (National Institute of Diabetes and Digestive and Kidney Diseases/National Institutes of Health, United States); Concepcion Nierras^9^ (Juvenile Diabetes Research Foundation International, United States); Elizabeth Thomson^8^ (National Human Genome Research Institute/National Institutes of Health, United States).

**Other Contributors:** David Altshuler (Whitehead Institute for Biomed Research, United States); Kinman Au^14^ (Medical College of Georgia, United States); Steve Bain^14^ (University of Wales Swansea, United Kingdom); Lisa Barcellos^13^ (University of California at Berkeley, United States); Sandra Barral^13^ (Rockefeller University, United States); Tim Becker^13^ (Karolinska Institutet, Sweden); Farren Briggs^13^ (University of California at Berkeley, United States); Paola Bronson^13^ (University of California at Berkeley, United States); Mark Daly^7,13^ (Massachusetts General Hospital, United States); Paul de Bakker^13^ (Massachusetts General Hospital, United States); Panos Deloukas^13^ (Wellcome Trust Sanger Institute, United Kingdom); Bernie Devlin^13^ (University of Pittsburgh, United States); Morten Chrisoph Eike^13,14^ (Institute of Immunology, Norway); Leigh Field^14^ (University of British Columbia, Canada); Stacey Gabriel (Broad Institute of MIT and Harvard, United States); Nikhil Garge^14^ (Medical College of Georgia, United States); Silvana Gaudieri^13^ (Murdoch University, Australia); Ben Goldstein^13^ (University of California at Berkeley, United States); Clara Gorodezky (INDRE SSA, Mexico); Sara Hamon^13^ (Rockefeller University, United States); Chungsheng He^13^ (Rockefeller University, United States); Joanna Howson^4,13,14^ (University of Cambridge, United Kingdom); Keith Humphreys^13^ (Karolinska Institutet, Sweden); Ian James^13^ (Murdoch University, Australia); Mark Lathrop^13^ (Centre National de Genotypage, France); Benedicte Alexandra Lie^13^ (University of Oslo, Norway); Dawei Li^13^ (Rockefeller University, United States); Steven Mack^13^ (Roche Molecular Systems, United States); Ralph McGinnis^13^ (Wellcome Trust Sanger Institute, United Kingdom); Elizabeth McKinnon^13^ (Murdoch University, Australia); William McLaren^13^ (Wellcome Trust Sanger Institute, United Kindgom); David Nolan^13^ (Murdoch University, Australia); Marita Olsson^13^ (Karolinska Institutet, Sweden); Jurg Ott^13^ (Rockefeller University, United States); David Owerbach (Baylor College of Medicine, United States); Chris Patterson^14^ (Queen’s University Belfast, United Kingdom); Robert Podolsky^14^ (Medical College of Georgia, United States); Patricia Ramsay^13^ (University of California at Berkeley, United States); Venkatesh Rangantah^13^ (Wellcome Trust Sanger Institute, United Kingdom); Neil Risch^13^ (University of California at San Francisco, United States); Kjersti Skjold Ronningen^14^ (Norwegian Institute of Public Health, Norway); Xiarong Shao^13^ (University of California at Berkeley, United States); Richard Single^13^ (University of Vermont, United States); Michael Steffes^5^ (University of Minnesota, United States); Glenys Thomson^13^ (University of California at Berkeley, United States); Ana Maria Valdes^5,13^ (Lartech, Italy); Claire Vandiedonck^13^ (Wellcome Trust Centre for Human Genetics, United Kingdom); Pam Whittaker (Wellcome Trust Sanger Institute, United Kindgom); Qingrun Zhang^13^ (Beijing Institute of Genomics, China).

**Study roles:** ^1^Steering Committee, ^2^Autoantibody Laboratory, ^3^HLA Genotyping Laboratory, ^4^Network DNA Repository, ^5^Quality Control Committee, ^6^Access Committee, ^7^Bioinformatics Committee, ^8^Ethical, Legal and Social Issues Committee, ^9^Molecular Technology Committee, ^10^Network Coordinators Committee, ^11^Phenotyping and Recruitment Committee, ^12^Publications and Presentations Committee, ^13^MHC Fine Mapping Working Group, ^14^Rapid Response Working Group, ^15^Network Principal Investigator.
